# Silent challenge: Early kidney transplant failure and cardiomyopathy

**DOI:** 10.1002/ccr3.6421

**Published:** 2022-10-11

**Authors:** Raquel Santana‐Estupiñán, Francisco Valga, María del Val Groba Marco, Juan Carlos Quevedo‐Reina, David Bongiovanni, José Carlos Rodríguez‐Pérez

**Affiliations:** ^1^ Nephrology Department Hospital Universitario de Gran Canaria Doctor Negrín Las Palmas de Gran Canaria Spain; ^2^ Cardiology Department Hospital Universitario de Gran Canaria Doctor Negrín Las Palmas de Gran Canaria Spain; ^3^ Pathology Department Hospital Universitario de Gran Canaria Doctor Negrín Las Palmas de Gran Canaria Spain

**Keywords:** cardiomyopathy, endomyocardial biopsy, kidney transplant, primary hyperoxaluria

## Abstract

Differentiation of hypertrophic cardiomyopathy phenotypes is challenging but crucial for appropriate management. We report a case of myocardial oxalate deposition as an infrequent cause of infiltrative cardiomyopathy.

## Graphical abstract

Cardiac remodeling occurs frequently in hypertensive patients with chronic kidney disease. However, other diseases with myocardial involvement should be considered. This clinical image shows the importance of the endomyocardial biopsy.

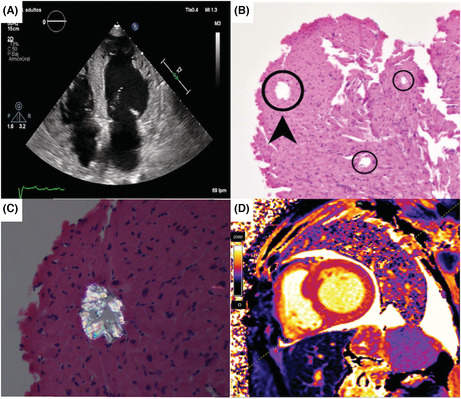

We present a 57‐year‐old woman with arterial hypertension since 2008 adequately controlled, and chronic kidney disease of unknown etiology undergoing dialysis since 2014. Pretransplant cardiac evaluation observed left ventricular (LV) hypertrophy attributed to hypertensive cardiomyopathy and normal coronary angiography. She received cadaveric kidney transplant (KT) in 2019 and presented early renal failure. Kidney biopsy revealed tubulointerstitial nephropathy due to oxalate crystals, and genetic study confirmed primary hyperoxaluria type 1. Pyridoxine and daily on‐line hemodialysis was initiated.

Echocardiogram showed LV dysfunction and severe wall thickness increase with granular texture (Figure 1A), right ventricular wall thickening, biatrial enlargement and restrictive mitral Doppler signal.^1^ Endomyocardial biopsy (EMB) demonstrated rosette‐like calcium oxalate crystals in myocardial fibers (Figure 1B,C) and interstitial fibrosis. Myocardial T1 mapping in cardiac magnetic resonance (CMR) showed diffuse fibrosis, dilated LV, and pleural and pericardial effusion (Figure 1D). Right heart catheterization revealed postcapillary pulmonary hypertension.

**FIGURE 1:**
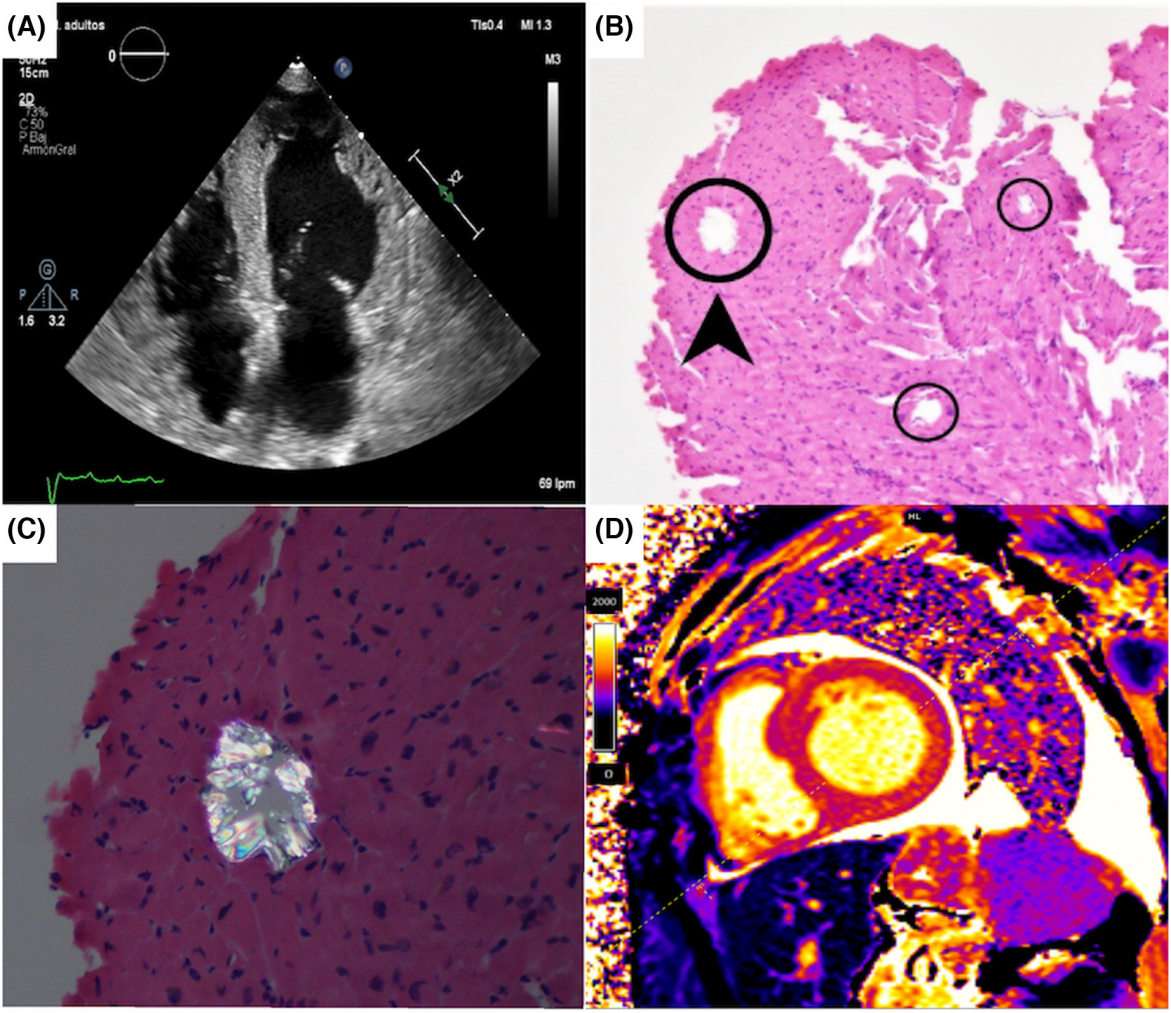
(A) Two‐dimensional 4‐chamber view during diastole. Symmetrical left and right ventricular wall thickness with echo‐dense speckled reflection. (B) Endomyocardial biopsy showing numerous oxalate crystals (circles). Hematoxylin and eosin (H‐E) × 40. (C) Endomyocardial biopsy showing oxalate crystal viewed under polarized light (H‐E × 200). (D) Non‐contrast T1 mapping (CMR), short axis orientation. Increase myocardial native T1 value. Note presence of pericardial and pleural effusion.

End‐stage oxalosis cardiomyopathy was made after KT failure.^2^ Combined liver‐kidney transplantation was not allowed due to operative risks. Lumasiran and heart failure treatment was started with a significant improvement of LV function (LV ejection fraction from 38% to 67%). She continues on dialysis.

Multidisciplinary approach and EMB was essential in the diagnosis of myocardial thickening.

## AUTHOR CONTRIBUTIONS

RS, FV, MG, and JQ treated the patient and wrote the manuscript. All authors contributed to manuscript revisions, approved the final version of the manuscript, and agree to be held accountable for the content therein.

## CONFLICT OF INTEREST

The authors have no conflict of interest to disclose.

## CONSENT

Written consent for publication was obtained from the patient and is available upon request.

## Data Availability

Data openly available in a public repository that issues datasets with DOIs.
